# Dynamically Tunable Exciton‐Photon Coupling via Multi‐Physics Field in Transition Metal Dichalcogenide Microcavities

**DOI:** 10.1002/advs.77179

**Published:** 2026-08-15

**Authors:** Jingwen Zhang, Wenqi Qian, Haiyi Liu, Guangyi Tao, Hai Liu, Yifan Zhang, Pengfei Qi, Zheyu Fang

**Affiliations:** ^1^ Institute of Modern Optics National Key Laboratory of Semiconductor Laser Nankai University Tianjin China; ^2^ Tianjin Key Laboratory of Micro‐Scale Optical Information Science and Technology Nankai University Tianjin China; ^3^ School of Physics, State Key Lab for Mesoscopic Physics, and Collaborative Innovation Center of Quantum Matter Peking University Beijing China; ^4^ Academy for Advanced Interdisciplinary Studies Nankai University Tianjin China

**Keywords:** exciton‐photon coupling, exciton‐polariton, light‐matter interaction, tunable microcavity

## Abstract

Atomically thin transition‐metal dichalcogenides (TMDs) host tightly bound excitons with large oscillator strengths, making them ideal for studying light‐matter interactions and polaritonic devices. When integrated with optical microcavities, TMD monolayers and van der Waals heterostructures enable engineering of the local density of optical states (LDOS), giving rise to Purcell‐enhanced emission in the weak coupling regime and exciton‐polaritons in the strong coupling regime. Practical polariton devices require dynamic, reversible control of exciton‐photon detuning and coupling strength, as these parameters determine Hopfield coefficients, dispersion, linewidth, relaxation dynamics, and nonlinear response. Unlike static microcavities, dynamic control enables exciton‐polaritons to adapt to diverse operating conditions, allowing real‐time functional switching and precise performance optimization. This strategy overcomes the inherent limitations of static control, such as limited functionality and adaptability. Recent advances have established several effective tuning strategies, including piezoelectric, electrical, thermal, and all‐optical approaches, enabling control over polariton states and functions including energy tuning, polarization/valley‐selective responses, and ultrafast optical modulation. This review summarizes these dynamic control strategies in microcavities and discusses key challenges and future directions toward scalable, stable, on‐chip integrated polariton platforms.

## Graphical abstract

Dynamic microcavities provide reversible control over exciton‐photon coupling in two‐dimensional TMDs through piezoelectric, electrical, thermal, and all‐optical tuning. By reshaping detuning, Rabi splitting, and polariton composition, these strategies enable adaptable polaritonic functions, including optical switching, polariton lasing, and reconfigurable on‐chip devices, toward integrated excitonic optoelectronics.

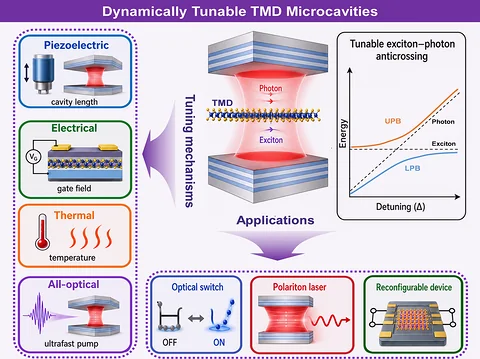

## 1 Introduction

Excitons, the hydrogen‐like bosonic quasiparticles of electron–hole pairs bound by Coulomb attraction, dominate the optical and electrical properties of most semiconductors. Their central role in materials science and solid‐state physics has spurred intensive efforts to harness excitons for low‐energy, ultrafast optoelectronic applications [1, 2, 3, 4]. Despite these advances, excitonic device development remains hindered because exciton binding energies in conventional quantum wells are typically below the room‐temperature thermal energy (∼26 meV). In contrast, reduced dielectric screening and enhanced quantum confinement in 2D TMDs give rise to excitons with binding energies up to 500 meV, which remain stable at room temperature and dominate the optical response [5, 6, 7, 8]. Upon thinning to the monolayer limit, an indirect‐to‐direct bandgap transition occurs, enhancing radiative recombination and photoluminescence (PL) quantum yield [9, 10, 11]. Furthermore, excitons in 2D TMDs exhibit valley polarization, tunability (via strain, electric field, etc.), and diverse excitonic complexes (e.g., trions, dark excitons, biexcitons), offering new degrees of freedom for quantum control and functional device engineering [10, 12, 13, 14, 15, 16, 17, 18, 19, 20, 21, 22, 23].

Precisely controlling excitonic dynamics is essential for practical applications, and various modulation strategies have been developed. External field modulation, such as microbubble‐induced strain [24] and applied constant magnetic field [25], has become a mainstream approach. However, most external field approaches modulate only exciton energies or populations; dynamic and reversible control of exciton‐photon coupling remains challenging. Optical microcavities manipulate exciton behavior via photon confinement and field enhancement, and their integration with TMDs has attracted increasing interest [26]. Currently, the mainstream optical microcavity structures include Fabry‐Pérot (FP) cavities [27, 28, 29], plasmonic nanocavities [30, 31, 32, 33], and photonic crystals [34, 35, 36]. When integrated with excitons in 2D TMDs, strong coupling between excitons and cavity photons forms unique hybrid light‐matter quasiparticles known as exciton‐polaritons [37], which exhibit exceptional characteristics such as low‐threshold emission and ultrafast response [27]. Conventional static microcavities offer fixed detuning, fixed coupling states, and limited functionality, failing to meet the requirements for switching, modulation, and performance optimization in practical devices [26]. Therefore, dynamic, reversible, and in situ control is crucial for transitioning such systems toward practical applications [38]. Unlike external field modulation, microcavities offer the unique advantage of directly manipulating the exciton‐photon coupling state. For example, a gate‐voltage‐tunable distributed Bragg reflector (DBR) microcavity enables switching of exciton‐polariton states in a MoS_2_ monolayer, with the lower polariton branch (LPB) and upper polariton branch (UPB) emerging under electrical control [39]. Moreover, embedding a MoSe_2_ monolayer in a tunable microcavity suppresses valley pseudospin loss via cavity‐modified exciton relaxation; the resulting valley polarization of polaritons is significantly enhanced and depends on the exciton‐photon detuning [40]. Notably, coupling TMDs to tunable microcavities enables precise control of exciton‐photon coupling, laying the groundwork for low‐power, high‐speed, and integrated optoelectronic devices.

**FIGURE 1:**
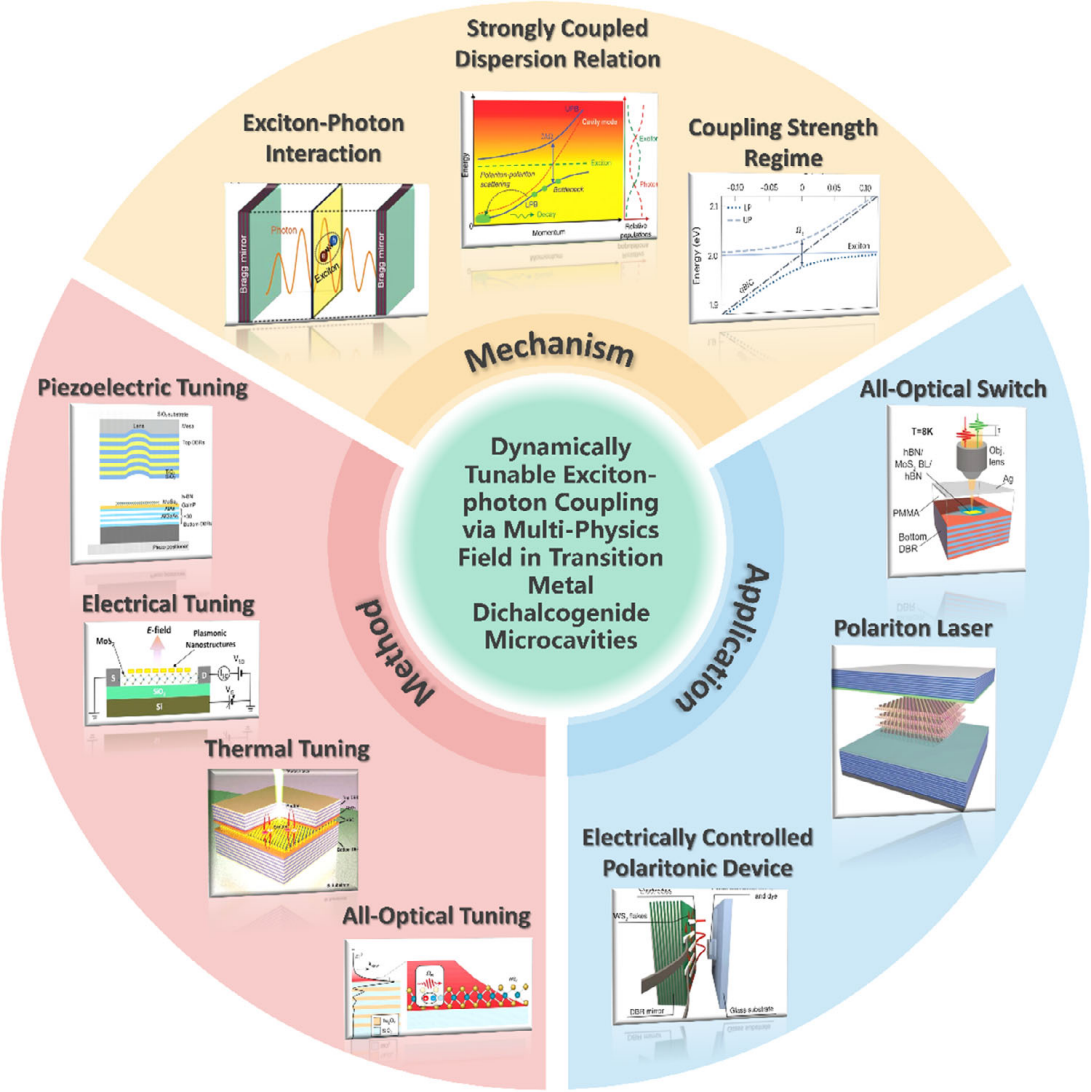
Schematic diagram of the mechanism, method, and application of dynamic modulation of 2D TMD exciton properties by optical microcavities [28, 34, 41, 42, 43, 44, 45, 46].

Although dynamic tuning is of critical importance for polaritonic devices, most existing reviews have primarily focused on exciton physics, static optical microcavity, or polariton condensation. Comprehensive comparisons of different dynamic modulation strategies from physical mechanisms to applicable scenarios remain scarce. Therefore, this review consolidates the previously scattered fields of exciton modulation, polaritonics, and microcavity design into a unified framework of dynamically tunable exciton‐photon coupling. Here we systematically benchmark four reversible control approaches across three perspectives of physical mechanisms, tunability, and resulting functionalities, charting a targeted roadmap toward low‐power, high‐speed on‐chip TMD polaritonic devices. Figure 1 outlines the structure of this paper. Section 2 describes the physical models of exciton‐cavity coupling. Section 3 reviews recent efforts to modulate excitonic properties in 2D TMDs using optical microcavities via multiple approaches (piezoelectric, electrical, thermal, and all‐optical), all of which have been shown to achieve precise tuning of excitonic properties. Section 4 discusses the potential applications of microcavity‐mediated dynamic modulation of TMD excitons, including all‐optical switches and polaritonic devices, offering insights for developing next‐generation excitonic optoelectronics. Finally, we discuss current challenges and future directions for microcavity‐based dynamic modulation of excitons in 2D TMDs.

## 2 Weak Coupling and Strong Coupling

The coupling between excitons and microcavity photons essentially constitutes a modulation process of light‐matter interaction. In 2D TMDs, high‐binding‐energy excitons provide the material platform, while optical microcavities modify exciton radiative dynamics and energy levels by confining the optical field and enhancing the LDOS. Light‐matter interaction in optical microcavities can occur in either the weak coupling or strong‐coupling regime depending on the strength of the interaction. The strong coupling regime is reached when the exciton‐photon coupling rate exceeds the decay rates of both the cavity photon and the exciton. This requires completing more than one round of energy exchange between excitons and photons before the system's coherence is fully disrupted. This implies that strong coupling is achieved when the exciton‐photon coupling strength *g* is larger than the linewidth of the uncoupled excitons (γ_*X*_) and photons (γ_*ph*_). This condition can be expressed as

(1) $$2g^{2}>\gamma_{X}^{2}+\gamma_{ph}^{2},$$

**FIGURE 2:**
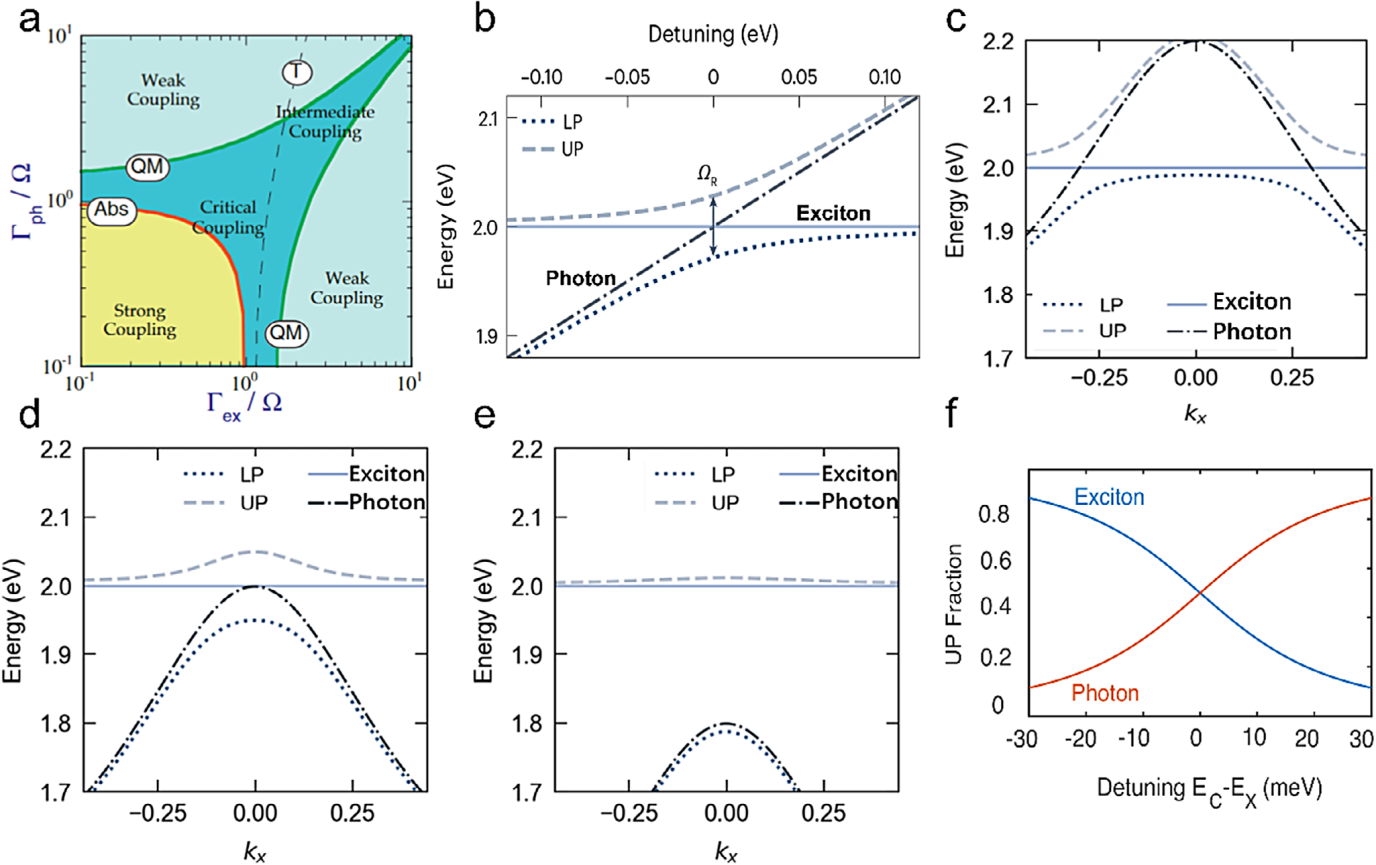
(a) Diagram of the different coupling regimes (Reprinted/ adapted with permission [50] 2005, John Wiley and Sons). (b) The UPB and LPB as a function of detuning. Angular dispersions of the LPB and UPB in microcavities exhibiting positive (c), zero (d), and negative (e) detunings relative to the exciton (Reprinted/ adapted with permission [46] 2025, Springer Nature). (f) The exciton and the photon fraction of the UPB as a function of detuning (Reprinted/ adapted with permission [51] 2021, Springer Nature).

Figure 2a presents the phase diagram of exciton‐cavity coupling. When the inequality above holds, the system is in the strong coupling regime. Conversely, when $4g^{2}<{\left(\gamma_{X}-\gamma_{ph}\right)}^{2}$, the system is in the weak coupling regime, with the region between them corresponding to the intermediate coupling regime [47, 48]. In the weak coupling regime, the interaction strength is insufficient to overcome the dissipative losses of the exciton and cavity mode, preventing coherent energy exchange between them. Excitons and photons retain independent quantum states without forming a coherent superposition, and the interaction is primarily manifested in the modification of exciton radiative behavior by the cavity field. The Purcell effect quantitatively describes this process [49]. Accordingly, weak‐coupling modulation depends primarily on cavity photonic parameters and manifests as changes in emission rate, directionality, and quantum yield, rather than the formation of new dispersion branches.

In the strong coupling regime, one can create hybrid light‐matter quasiparticles called exciton‐polaritons, which are superpositions of excitons and cavity photons. The Hamiltonian for a strongly coupled exciton‐cavity system is [52]

(2) $$H_{k}=E_{X}\left(k\right)b_{k}^{†}b_{k}+E_{ph}\left(k\right)a_{k}^{†}a_{k}+gb_{k}^{†}a_{k}+ga_{k}^{†}b_{k},$$

where*E_ph_* (*k*) and *E_X_* (*k*) are the energies of cavity photons and excitons at wavevector *k*; *a*†/*a*and *b*†/*b*are the creation/annihilation operators of photons and excitons, respectively. The coupling factor *g* is [52]

(3) $$g=\frac{1}{2}\sqrt{f}\int u_{ph}\left(r\right)u_{X}\left(r\right)dr,$$

Here, *f* is the oscillator strength, ∫*u_ph_* (*r*)*u_X_* (*r*)*dr* is the overlap integral between the electromagnetic field *u_ph_* (*r*) and the exciton wave function *u_X_* (*r*). A hallmark of strong coupling is vacuum Rabi splitting (*Ω*), where the energy levels split into the UPB and LPB, as shown in Figure 2b. Diagonalizing the Hamiltonian gives the two branch energies [52]

(4) $$E_{UPB,LPB}\left(k\right)=\frac{E_{ph}\left(k\right)+E_{X}\left(k\right)}{2}\pm \sqrt{\Delta^{2}+g^{2}},$$

where Δ = [*E_ph_* (*k*) − *E_X_* (*k*)]/2 is the exciton‐photon detuning between photons and excitons. At Δ = 0, the splitting is minimal and equals *Ω*,

(5) Ω=2g,

Figure 2c–e illustrate the splitting for positive, zero, and negative detuning, respectively. In the strong coupling regime, excitons and photons undergo quantum state hybridization to form exciton‐polaritons, which are essentially linear superpositions of photonic and excitonic states. The coefficients of the excitonic and photonic components, known as Hopfield coefficients, determine the exciton and photon fractions of a polariton state. For strongly coupled excitons and photons, the single‐polariton operator is [37, 38]

(6) $$\Psi_{pol}=C_{ph}a+C_{X}b,$$

where*C_ph_* and *C_X_* are the Hopfield coefficients, corresponding to the weights of the photonic and excitonic components in the polaritons, respectively. Physically, |*C_ph_* |^2^ is the photonic fraction of the polariton; a larger value means the exciton‐polariton is more photon‐like.|*C_X_* |^2^ is the excitonic fraction; a larger value means it is more exciton‐like. Figure 2f shows the variation of the excitonic and photonic proportions corresponding to the UPB as a function of detuning [37, 47, 48, 52]. Importantly, tuning the detuning provides a direct handle to reshape the polariton composition, which in turn governs key observables such as linewidth, relaxation dynamics, nonlinear response, and polarization/valley characteristics.

## 3 Dynamic Control of TMD Exciton‐Polaritons

As the core platform for controlling the TMD exciton properties, the tunable optical microcavities constitute a very interesting and versatile platform for the investigation of light‐matter interactions. Unlike static microcavities, which offer fixed parameter settings, dynamic control enables in situ, reversible, and real‐time modulation. This dynamic tunability is essential for device functions such as switching, modulation, and performance optimization. Currently, the mainstream tuning approaches encompass four categories: piezoelectric tuning, electrical tuning, thermal tuning, and all‐optical tuning. Piezoelectric tuning provides broad and precise control over exciton‐photon detuning; electrical tuning is more compatible with active devices and enables in situ reversibility; thermal tuning offers a simple route for detuning scans but is intrinsically slow and global; whereas all‐optical tuning is ultrafast and contact‐free but often transient and pump‐dependent.

### 3.1 Piezoelectric Tuning

Piezoelectric tuning uses piezoelectric actuators to dynamically adjust the cavity length and thus control the detuning between the cavity resonance and the exciton energy. This mechanism allows in situ reversible tuning of polariton dispersion, composition, linewidth, and relaxation dynamics. For the FP microcavity, the cavity resonance is set by the longitudinal condition [55]

(7) $$m\frac{\lambda_{c}}{2}=n_{eff}\cdot L_{eff},$$

where *m* is the longitudinal mode index, *λ_c_* is the resonant wavelength, *n_eff_* and *L_eff_* represent the effective refractive index and cavity length, respectively. Thus, changing the cavity length tunes the resonance condition. Typically, the mirror is installed on a motorized piezo to form an open cavity. The mirror separation can be controlled with sub‐nanometer precision using piezoelectric actuators, providing a simple mechanism for tuning the cavity resonances to any desired wavelength. This method allows for in situ, reversible tuning without chemical doping or permanent material alteration. The cavity resonance energy *E_ph_* is inversely proportional to the cavity length. By varying the detuning between the exciton resonance and the cavity mode, piezoelectric tuning directly controls the polariton composition, dispersion curvature, linewidth, and relaxation dynamics. At zero detuning (*E_ph_ = E_X_*), the anticrossing of the UPB and LPB is most pronounced. Away from resonance (*E_ph_≠E_X_*), the polariton states lose their mixed character and asymptotically approach the uncoupled exciton and photon modes, leading to pronounced changes in dispersion and relaxation behavior.

**FIGURE 3:**
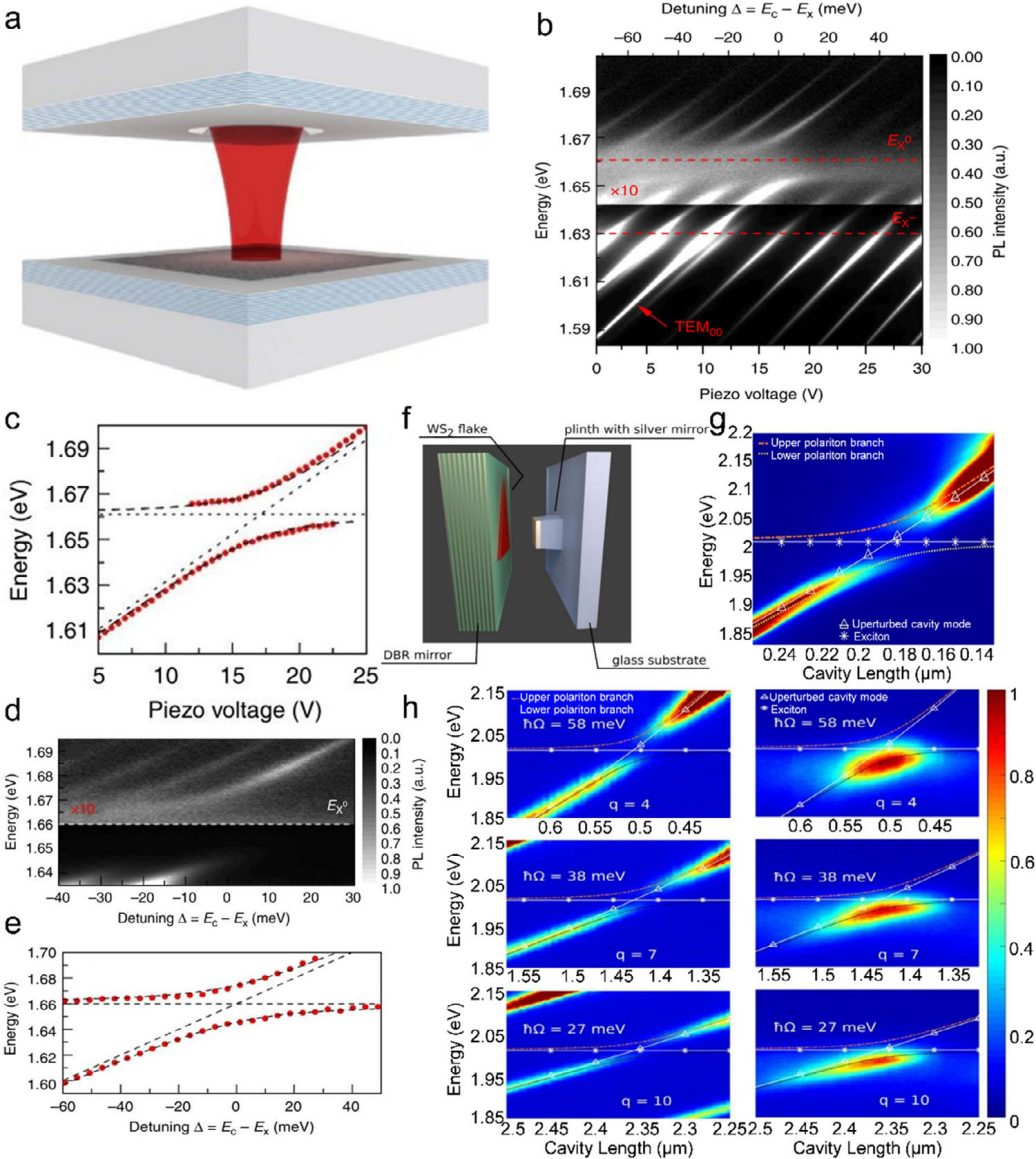
(a) Schematic of the tunable hemispherical cavity with an embedded MoSe_2_ heterostructure. (b) A clear anticrossing behavior in PL is observed between the discrete cavity mode energies and the neutral exciton energy at 4.2 K. The longitudinal resonance is labeled TEM_00_. (c) The UPB and LPB are fitted for the longitudinal mode with *Ω* = 20 meV. (d) The double quantum well structure shows an anticrossing behavior between the neutral exciton and discrete cavity modes at 4.2 K. (e) A fit to the peak position as a function of detuning (Reprinted/ adapted with permission [53] 2015, Springer Nature). (f) Schematics of cavity setup. (g) Transmission spectra as the cavity length is swept, showing typical polariton dispersion. The white continuous lines show the energy of the unperturbed cavity mode (triangles) and the exciton (stars). The energy of the coupled system, namely the two polariton branches, is shown with colored dashed lines. (h) Transmission (left) and PL (right) spectra as cavity length is swept, traversing different longitudinal modes. The lines show the dispersion of the uncoupled (white) and coupled system (color) (Reprinted/ adapted with permission [54] 2016, Springer Nature).

Based on this strategy, Dufferwiel et al. [53] first integrated a MoSe_2_/hexagonal boron nitride (hBN) heterostructure in a tunable microcavity, as illustrated in Figure 3a. The cavity modes are tuned through the neutral exciton resonance by reducing the mirror separation by applying a d.c. voltage to the bottom *z*‐nanopositioner. Reflection and PL spectroscopy (Figure 3b) revealed a clear anticrossing between the tunable cavity mode and the MoSe_2_ exciton energy. Fitting the UPB and LPB peak energies of the longitudinal mode with a coupled oscillator model (dashed lines in Figure 3c) yields *Ω* = 20 meV for a MoSe_2_ monolayer. Furthermore, they investigated the MoSe_2_/hBN/MoSe_2_ double quantum wells in the open microcavity. A fit to the polariton peak energies using the coupled oscillator model reveals an increased *Ω* of 29 meV (Figure 3d,e). This pronounced anticrossing confirms robust strong coupling in monolayer TMD microcavities. Building on this work, Flatten et al. [54] coupled a WS_2_ monolayer to an open FP cavity (Figure 3f). By varying the cavity length with a piezo micro‐actuator, stable cavity modes interacting strongly with the WS_2_ can be obtained, resulting in a maximal *Ω* of 70 meV (Figure 3g). Furthermore, variations in cavity length and *Ω* exert a significant impact on the population dynamics. Under non‐resonant pumping conditions, the polariton population increases linearly with increasing *Ω*: only the LPB is effectively populated, while the UPB remains unpopulated (Figure 3h). This further demonstrates the effective modulation of TMD exciton‐polariton behaviors by this tuning method.

**FIGURE 4:**
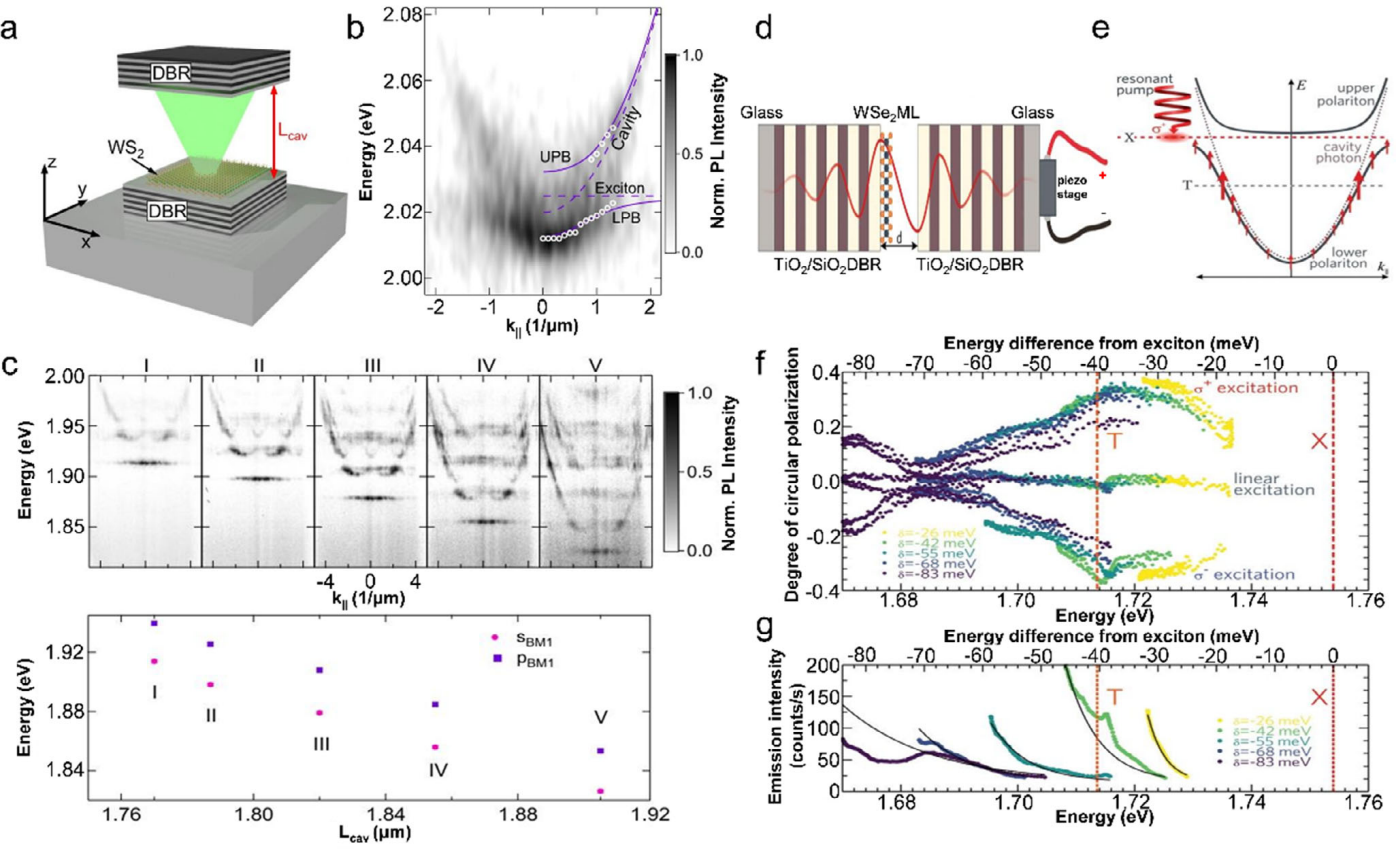
(a) Sketch of the open cavity device, composed of two DBRs embedding an air gap. (b) Momentum‐resolved PL spectrum of the polariton emission for the cavity length of approximately 2.2 µm, under weak excitation with a green, non‐resonant, continuous wave laser. (c) Top: Momentum‐resolved PL spectra of the 1D linear chain for increasing DBR separations from left to right. The PL map intensities are coded in a false color scale included on the right side of the panels. Bottom: Dependence of the s‐ (magenta circles) and p‐band (violet squares) energy vs. cavity length. The energy was tuned over 87.8 meV (s‐band) and 86.3 meV (p‐band) with a change of the DBR separation by 135 nm (Reprinted/ adapted with permission [56] 2021, Springer Nature). (d) Scheme of the open, tunable cavity. (e) Coupling of excitonic transitions to a cavity mode. The spin‐preserving polariton relaxation along the lower polariton branch is marked by red arrows. (f) The DOCP of the LPB collected at 5 different detunings, each excited with σ+, σ‐, and linear polarization. The energy position of trion T based on PL spectra is marked. (g) Emission intensity depending on the emission energy. Boltzmann distribution fits are marked with black lines with temperatures equal to: 171, 100, 72, 64, and 35 K, from the most negative detuning (Reprinted/ adapted with permission [57] 2019, RCS Pub).

In addition to modulating the exciton‐photon coupling strength, piezoelectric tuning also engineers the functional properties of exciton‐polaritons, including band structure, valley‐selective responses, and relaxation pathways. For band‐structure engineering, Lackner et al. [56] integrated a WS_2_ monolayer into a cavity incorporating a photonic lattice (Figure 4a). The two mirrors form an open cavity whose resonance is tunable by varying the DBR separation with nanometer precision. The dispersion relation of the polariton PL is shown in Figure 4b. Using a standard coupled oscillator model, they confirmed strong coupling with *Ω =* 19 meV and a tuning range exceeding 85 meV (Figure 4b,c). These results highlighted the ability of piezoelectric tuning to tailor polaritonic band structures in complex cavity architectures, offering a promising platform for exploring topological exciton‐polariton states. Piezoelectric tuning also manipulates valley‐dependent polariton properties. Król et al. [57] integrated a WSe_2_ monolayer into a tunable dielectric microcavity (Figure 4d), achieving a tuning range exceeding 80 meV. As depicted in Figure 4e, the broad tuning range enables exciton‐polariton modes to resonate with trions and low‐energy exciton transitions. When the exciton‐polariton energy resonates with trions, valley polarization increases significantly, with the degree of circular polarization (DOCP) approaching 40%. This enhancement is attributed to the substantially longer valley depolarization time of trions compared with neutral excitons. In contrast, when the exciton‐polaritons resonate with the low‐energy state (near 1.687 eV in Figure 4f), valley polarization drops sharply, even undergoing complete depolarization; concurrently, this is accompanied by a reduction in emission intensity (Figure 4g), which is attributed to resonance‐enhanced scattering into non‐radiative channels along with spin‐flip processes.

**FIGURE 5:**
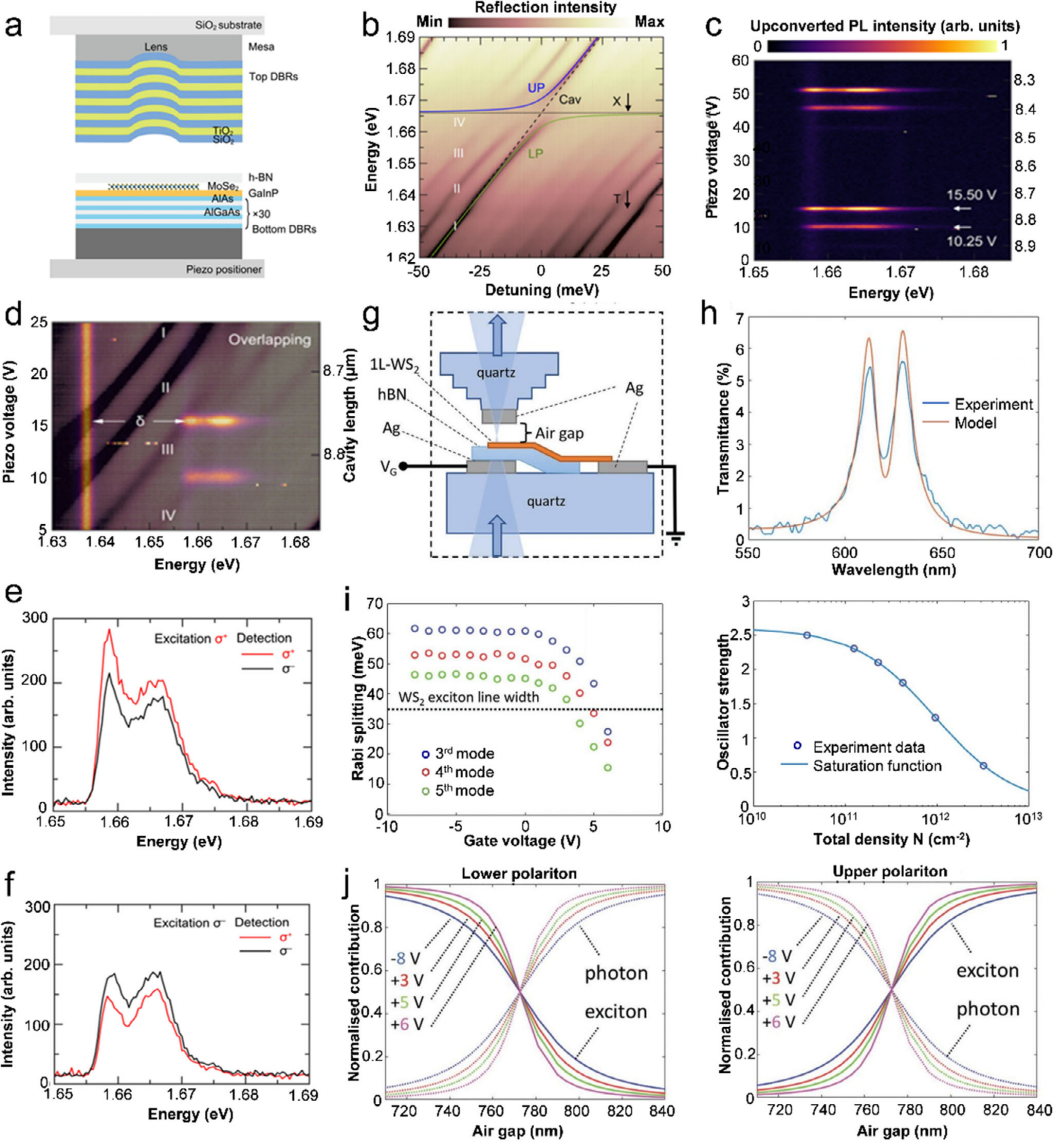
(a) Schematic diagrams of the open cavity system. (b) Reflection spectra recorded at different detuning. (c) Upconversion PL plotted in false color scale as a function of piezo voltage (cavity length), with the excitation energy of 1.638 eV. (d) Upconversion PL only occurs from polaritonic doublets (transverse modes III or IV), while the pump laser is resonant with the ground mode I or transverse mode II. *δ* is the upconverted energy from trions to exciton‐polaritons. (e) Circular polarization of upconversion emission with σ^+^ excitation and (f) σ^−^ excitation (Reprinted/ adapted with permission [42] 2025, Springer Nature). (g) Schematic of the tunable microcavity. (h) Experimental and calculated transmittance of a strongly coupled WS_2_ microcavity. Strong coupling was achieved for a cavity air gap of 787 ± 3 nm, which corresponds to the third‐order cavity mode. (i) Left panel: *Ω* as a function of the gate‐voltage. The three different cavity modes are shown as different colors: blue for the third mode, red for the fourth mode, and green for the fifth mode. Right panel: Oscillator strengths as a function of the total density of electrons *N*. (j) The Hopfield coefficients of the LPB (left) and UPB (right), respectively. The different colors correspond to different values of the gate‐voltage. Continuous lines correspond to the exciton contribution to the polariton bands. Dashed lines correspond to the cavity photon contribution (Reprinted/ adapted with permission [58], 2019, John Wiley and Sons).

Piezoelectric tuning can further tailor polariton relaxation pathways. Shan et al. [42] demonstrated efficient upconverted luminescence in an open microcavity containing a charged MoSe_2_ monolayer (Figure 5a). They coupled two cavity modes of the open cavity with electronic transitions of a MoSe_2_ monolayer, measuring reflection spectra recorded at different detuning (Figure 5b) and observing a cavity‐mediated double resonance condition for efficient upconversion: one mode weakly couples to trions, while the other is on resonance with excitons, yielding exciton‐polaritons through strong coupling (Figure 5c,d). The upconverted emission also preserves a finite valley polarization (Figure 5e,f), indicating that piezoelectric tuning can simultaneously manipulate relaxation dynamics and polarization‐selective optical responses.

Piezoelectric tuning is widely adopted in open‐cavity and precision spectroscopy studies, primarily owing to its high mechanical precision and low‐perturbation characteristics. Sub‐nanometer displacement control enables continuous adjustment of the cavity length, affording detuning scans with extremely high resolution. This nearly purely geometric tuning approach leaves the intrinsic electronic structure of the material largely intact. However, this method still suffers from notable bottlenecks in practical applicability. First, the mechanical stability of open‐cavity configurations constrains long‐term integration; micro‐vibrations and thermal drift directly compromise cavity‐length stability, rendering the system environmentally sensitive. Second, as an inherently macroscopic mechanical control strategy, piezoelectric tuning is difficult to integrate in high‐density chips, restricting its application potential in on‐chip multi‐channel devices. Furthermore, the tuning speed is constrained by the mechanical response frequency of piezoelectric materials, precluding high‐speed dynamic modulation. Further advances in microfabrication, cavity stabilization, and integrated actuation will help extend piezoelectric tuning from fundamental studies to polaritonic devices and reconfigurable photonic architectures.

### 3.2 Electrical Tuning

An external electric field modulates exciton intrinsic parameters, including energy levels, oscillator strengths, and population distributions. This modulation alters the coupling conditions between exciton and cavity mode, specifically the exciton‐photon detuning and the coupling strength. Consequently, reversible switching between the strong and weak coupling regimes, reconstruction of polariton dispersion curves, and dynamic manipulation of nonlinear optical effects are enabled. Representative electrical modulation schemes include gate‐voltage control and the quantum‐confined Stark effect (QCSE) [59, 60].

Gate‐voltage control is widely used to electrically modulate exciton‐polaritons. This method typically employs a field‐effect transistor (FET) configuration. Applying an external gate‐voltage injects electrons and holes into TMDs, thus modulating the carrier concentration. These free carriers interact with excitons through two primary mechanisms. On the one hand, free carriers can bind with neutral excitons to form trions. Trions have different energy levels and oscillator strengths from neutral excitons. The formation of trions directly modifies the energy and transition characteristics of the excitonic system [14, 61, 62]. On the other hand, varying the carrier concentration modifies the dielectric environment and Fermi level. A change in the dielectric environment influences the exciton binding energy and transition dipole moment, while shifting the Fermi level modulates exciton occupation and transition probability via the Pauli blocking effect [6, 63, 64, 65]. Gate‐voltage controls polaritons by tuning the exciton oscillator strength and spectral weight, thus modifying the exciton‐photon coupling. For a single exciton resonance, the coupling strength is [66]

(8) $$g\propto \mu \sqrt{\frac{\omega_{c}}{2\varepsilon \varepsilon_{0}A}},$$

where, *µ* represents the excitation transition dipole moment, and *A* is the cavity area. Since *µ^2^* is proportional to the oscillator strength *f* [67]

(9) $$\Omega \propto \sqrt{f},$$

Thus, when the gate‐voltage weakens the neutral exciton oscillator strength via screening and Pauli blocking, *Ω* decreases accordingly. A more microscopic view shows that doping not only reduces the bare exciton oscillator strength but also redistributes spectral weights among multi‐body resonances. Consequently, the coupling between the neutral exciton, trion, and cavity mode is often reconfigured [66]. Continuously tuning the gate‐voltage provides dynamic control over the exciton‐cavity coupling state. This approach thus provides an effective means of manipulating light‐matter interactions. Henry et al. [58] combined a FET with a tunable optical microcavity (Figure 5g), realizing precise electrical modulation of light‐matter coupling at room temperature. When the cavity mode resonates with neutral excitons of WS_2_, *Ω* reaches 60 meV (Figure 5h). As illustrated in Figure 5i, applying a gate‐voltage from −8 to +8 V increases the free electron concentration from 0 to 3.2 × 10^1^ ^2^ cm^−^^2^. The resulting Coulomb screening effect reduces the exciton oscillator strengths, causing *Ω* to gradually decrease from its saturated maximum to zero. This enables a continuous transition from strong to weak coupling. The Hopfield coefficients (Figure 5j) show that as the gate‐voltage becomes more positive, the LPB becomes more photonic, while the UPB becomes more excitonic. These observations demonstrate electrically tunable exciton‐photon decoupling, a key strategy for tunable optoelectronic devices. Furthermore, Sidler et al. [62] embedded a charge‐tunable MoSe_2_ monolayer in an open microcavity and observed Fermi polaron polaritons, which arise from the interaction between carriers and excitons. Electrostatic doping precisely controls the carrier density, thereby tuning the Fermi polaron characteristics. This work provides a new paradigm for exploring polariton physics in multi‐body interactions.

**FIGURE 6:**
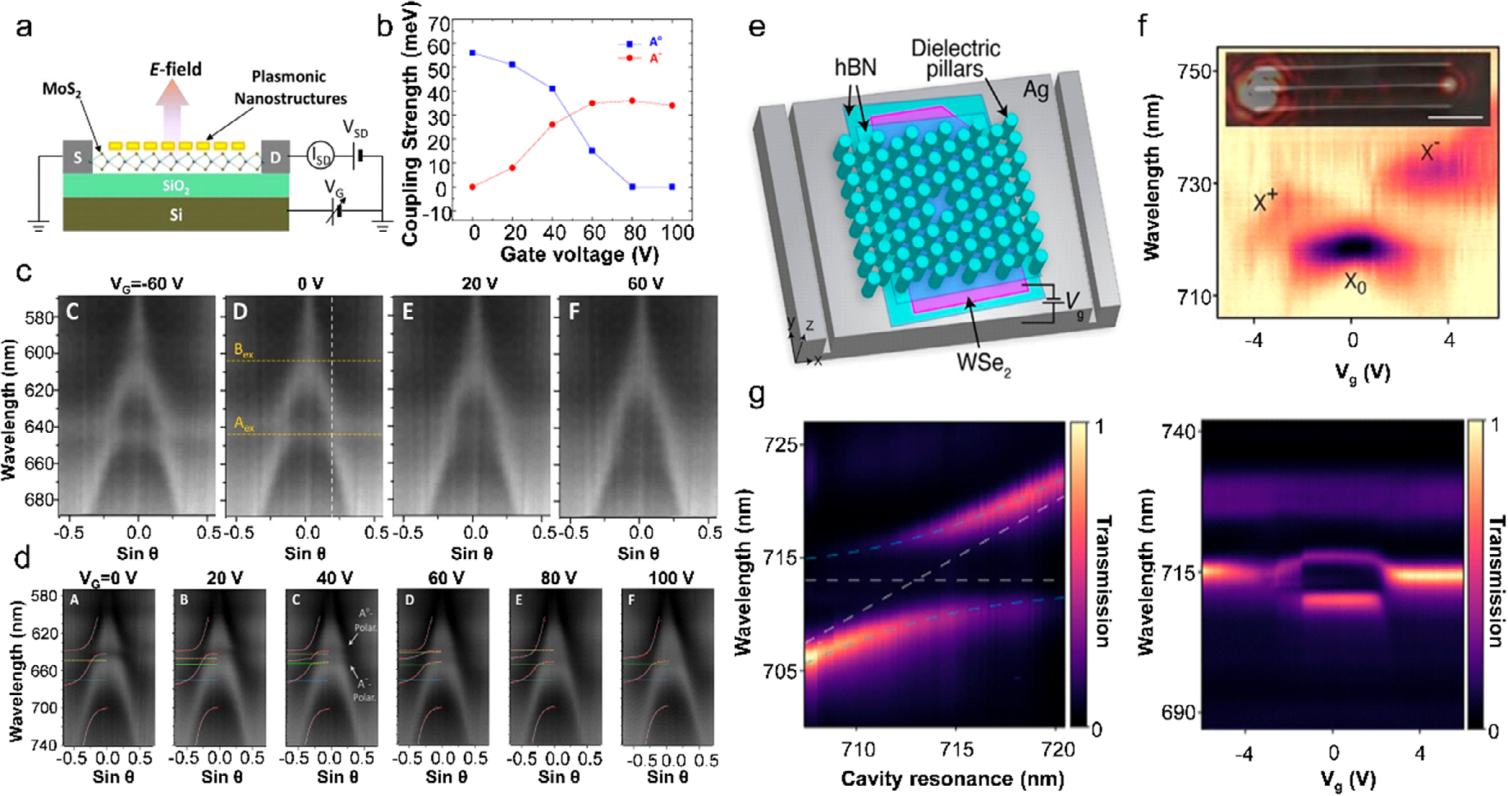
(a) Schematic diagram of the MoS_2_ monolayer with a plasmonic lattice integrated in an FET device geometry for carrier injection and depletion. (b) Calculated coupling strengths for neutral excitons and trion plasmon polariton modes as obtained from the coupled oscillator model are plotted as a function of the gate‐voltage. (c) Angle‐resolved differential reflectance spectra measured at different gate‐voltages. (d) Angle‐resolved contour plots and the corresponding coupled oscillator model fits are presented at different gate‐voltages. The position of neutral excitons and trions, and localized surface plasmon resonances are indicated as yellow, green, and blue‐dashed lines, respectively, in (A to F) (Reprinted/ adapted with permission [43] 2025, Springer Nature). (e) Schematic of the plasmonic crystal cavity between two trenches etched in the silver that serve as in‐coupling and out‐coupling structures for SPPs. (f) TiO_2_ waveguide‐assisted SPP transmission spectra through a WSe_2_ monolayer collected as a function of gate‐voltage. The spectra are collected at *T* = 4 K, and the absorption feature denoted by X_0_ (X^+^ and X^−^) indicates the neutral (charged) exciton species when the Fermi level is inside (outside) the bandgap region. Inset: Optical image overlaid on a scanning electron microscope image, demonstrating in‐coupling (left) and out‐coupling (right) at the ends of the TiO_2_ tapered waveguide. (g) Left panel: SPP transmission spectra of the plasmonic crystal cavity display an avoided crossing as the cavity is tuned in situ through the exciton absorption resonance. The white dashed lines show the uncoupled exciton and plasmonic crystal cavity mode, whereas the dashed blue curves correspond to theoretical fits of the hybrid states using a coupled oscillator model. Right panel: Cavity transmission as a function of gate‐voltage (Reprinted/ adapted with permission [74] 2019, American Chemical Society).

Gate‐voltage control also works well in plasmonic microcavities. Building on prior observations in hybrid MoS_2_‐plasmonic nanoantenna systems [68, 69, 70, 71, 72, 73], Lee et al. [43] demonstrated electrical control of exciton‐plasmon polariton coupling using plasmonic nanoresonators in a FET device (Figure 6a). Owing to the atomically thin nature of the TMD monolayer and the associated significantly reduced screening of the charged carriers, their optoelectronic properties can be controlled via electrostatic doping induced by the gate‐voltage. The coupling strength showed a clear dependence on doping concentration (Figure 6b). In the low‐doping regime, carrier depletion strengthened the A exciton‐plasmon coupling and increased the polariton mode splitting, yielding a distinct *Ω* (Figure 6c). As the carrier concentration increased, excitons became increasingly screened, leading to a reduction in oscillator strengths. Ultimately, the system switched from the strong to the weak coupling regime. They also found that trion resonances can couple strongly with lattice plasmons at high electron doping, forming trion‐plasmon polaritons (Figure 6d). which coupling characteristics could be tuned via electrostatic doping, providing a new avenue for the development of multi‐exciton polaritonic devices. Similarly, Dibos et al. [74] constructed a plasmonic crystal cavity system based on a WSe_2_ monolayer (Figure 6e). The tight confinement of the surface plasmon polaritons (SPPs) in the plasmonic cavity enables strong coupling between excitons and SPPs when the WSe_2_ exciton energy is resonant with the cavity mode, with *Ω* reaching up to 18 meV. Electrostatic doping provided dynamic control of the exciton‐plasmon coupling strength. Near zero gate‐voltage, neutral excitons dominated the plasmon absorption spectrum. When the gate‐voltage exceeded 2 V, the neutral exciton absorption weakened, and a broadened, redshifted trion absorption peak emerged (Figure 6f). This study realized the electrical switching of exciton‐plasmon strong coupling. As shown in Figure 6g, at gate‐voltages of 0 and 2.2 V, the transmission splits into two peaks as the WSe_2_ neutral exciton absorption is tuned onto resonance with the cavity. As the doping concentration is increased at larger bias voltages, the neutral exciton absorption is suppressed leading to a single peak, providing a promising platform for optoelectronic devices such as electrically tunable plasmonic modulators.

The QCSE is another key mechanism for electrical tuning [75]. It typically uses a vertical electric field configuration, where a TMD thin film sits between two electrodes. The external electric field shifts electrons and holes to opposite sides of the well, decreasing the overlap integral and modifying the exciton binding energy. The resulting exciton energy shift *∆E* is [75, 76]

(10) $$\Delta E=-\mu_{z}F_{z}-\beta_{z}F_{z}^{2},$$

where *F_z_* is the component of the electric field, *µ_z_* is the excitonic dipole moment, and *β_z_* is the excitonic polarizability along the *z*‐direction. Interlayer excitons (IXs), unique to TMD heterostructures, have out‐of‐plane electric dipoles that make them highly susceptible to QCSE modulation [77, 78, 79, 80]. For IXs, the influence of the QCSE can be expressed as [76]

(11) $$\Delta E_{IXs}=-\mu_{IXs}F_{z}=-e\cdot d\cdot F_{z},$$

**FIGURE 7:**
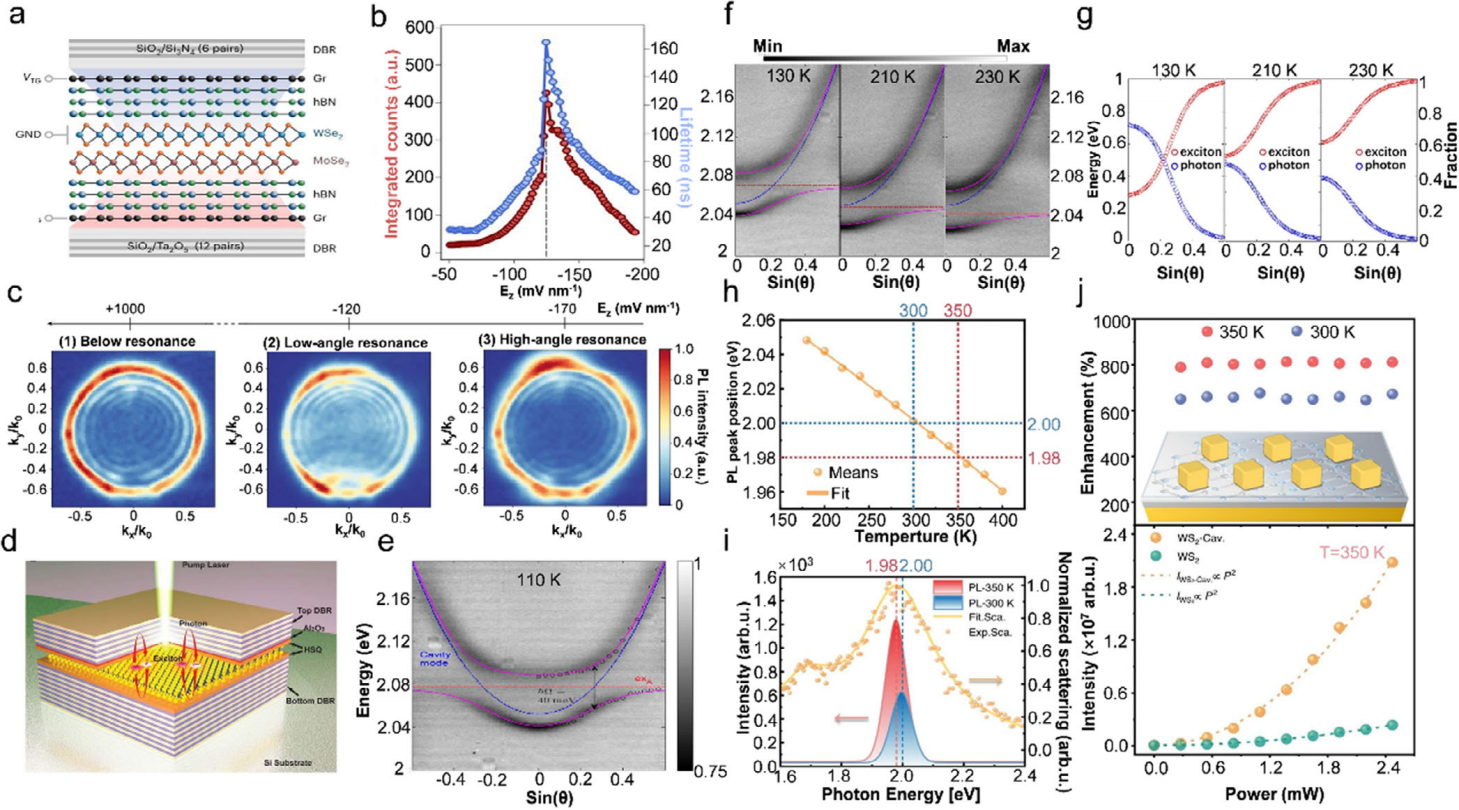
(a) Schematic of the device structure. (b) Total integrated IX PL intensity (red) and lifetime (blue) as a function of *E_z_*. (c) Momentum‐resolved IX emission obtained by back focal plane PL spectroscopy at electric field strengths of *E_z_* ≃ 100, −120, and −170 mV nm^−^^1^ (Reprinted/ adapted with permission [81] 2025, Springer Nature). (d) Schematic of the microcavity structure. (e) The reflectivity map at 110 K obtained by *k*‐space spectroscopy, where the horizontal axis represents the sine function of the incidence angles (*θ*) and the vertical axis is the photon energy. The gray color scale represents the reflectivity, with darker areas corresponding to lower reflectivity. (f) The *k*‐space reflectivity map at temperatures of 130, 210, and 230 K. As the temperature increases, the exciton (the red dashed line) redshifts from 2.072 to 2.042 eV, while the cavity photon dispersion (the blue dashed curve) does not change, creating various exciton‐photon detunings over this temperature range. The coupled oscillator model is calculated to fit the dispersions (magenta solid curves). (g) Hopfield coefficients of lower polariton states at three temperatures (Reprinted/ adapted with permission [28] 2017, American Physical Society). (h) Temperature‐dependent PL peak position for WS_2_ on Au/SiO_2_/Si. Yellow lines: fitted results by the Varshni equation. (i) Scattering spectrum (yellow dots) of the hybrid system and PL spectrum of the pristine monolayer WS_2_ at 350 K (red curve) and 300 K (blue curve) on the Au/SiO_2_/Si substrate. The measured scattering spectrum is fitted with Gaussian functions (yellow curve). (j) Excitation power‐dependent integrated upconverted emission intensity (down) and enhancement (top) for monolayer WS_2_ in the designed plasmonic cavity. The experimental results (scattering points) can be fitted well by the green and yellow dotted lines based on the power law formula *I = bP^α^*. In the illustration above, red (blue) dots represent the enhancement factor at 350 K (300 K). The inset is a schematic diagram of the plasmonic cavity structure (Reprinted/ adapted with permission [33] 2025, Springer Nature).

Applying an electric field controls the IXs energy (*E_IXs_*), thereby dynamically controlling the exciton‐photon detuning. Leveraging this property, Lopriore et al. [81] achieved electrostatic control over the coupling of dipolar excitons by applying a vertical electric field (*E_z_*) in a van der Waals heterostructure, composed of a WSe_2_ /MoSe_2_ bilayer hosting IXs and embedded in FP microcavities (Figure 7a). At *E_z_* ≈ −120 mV nm^−^^1^, the excitonic energy resonates with the cavity mode, resulting in a pronounced PL enhancement. Under resonant conditions, the IX PL intensity increased 50‐fold, and its lifetime increased 5‐fold (Figure 7b). The electric field modulates the momentum distribution of IXs: low‐momentum (low‐angle) emission dominates under resonance, whereas high‐momentum (high‐angle) emission prevails off resonance (Figure 7c). This modulation correlates with changes in the IXs diffusion length. Collectively, this work realizes electrically tunable coupling between IXs and cavity modes, establishing a new platform for investigating exciton condensates and electrically tunable exciton‐polaritonic devices. It also offers a strategy for controlling interlayer exciton‐polariton emission.

Electrical tuning has emerged as a highly promising approach for device applications owing to its inherent on‐chip integration compatibility and fast reversibility. Through gate‐voltage or vertical electric fields, one can simultaneously modulate the carrier density, exciton oscillator strength, and many‐body interactions, thereby enabling a continuous and controllable transition between strong and weak coupling. This multi‐parameter tuning capability makes it particularly suitable for FET‐type polariton devices, reconfigurable optical modulators, and photonic logic structures. However, its core bottleneck lies in the inherent conflict between electrical tunability and optical quality. First, high‐level carrier injection inevitably introduces Coulomb screening and free‐carrier absorption, which broaden the exciton linewidth and thus weaken the strong coupling condition. Second, interface traps and impurity charges introduce static disorder potentials that reduce the polariton coherence length. Furthermore, in multilayer heterostructures, the electric field distribution is often non‐uniform, resulting in a spatially inhomogeneous tuning response, which poses a challenge to the uniformity of large‐area devices. Looking ahead, advances in materials science and device engineering will make electrical tuning a key enabler of room‐temperature, ultralow‐power optoelectronic devices and multifunctional integrated systems for quantum information processing.

### 3.3 Thermal Tuning

TMD monolayers have Wannier‐Mott excitons with large binding energies that enable the tuning of the exciton part over a large temperature range. Temperature modulates exciton‐photon coupling primarily through thermally induced shifts of excitonic energy levels. The temperature dependence of the exciton energy follows the Varshni formula [82]

(12) $$E_{g}\left(T\right)=E_{g}\left(0\right)-\frac{\alpha T^{2}}{T+\beta },$$

where *E_g_* (0) represents the bandgap at 0 K, while *α* and *β* are fitting parameters specific to the material. This yields a continuously tunable exciton‐photon detuning, which controls the coupling regime. In a TMD monolayer, the excitons exhibit a clear temperature‐dependent shift. At low temperatures, weaker lattice vibrations reduce exciton‐phonon scattering, and excitons occupy higher energy levels. As the temperature increases, enhanced lattice vibrations strengthen electron‐phonon scattering, leading to a redshift of the excitonic energy levels [83]. In contrast, the cavity mode is almost independent of temperature. This differential thermal response enables thermal control of light‐matter interactions.

Liu et al. [28] introduced an efficient strategy to incorporate a WS_2_ monolayer into a compact all‐dielectric microcavity (Figure 7d). Here, they have demonstrated for the first time that the temperature dependence of the exciton resonance can be exploited to tune the exciton‐photon detuning. Within the temperature range of 110–230 K, the system achieves strong coupling, exhibiting anticrossing polariton dispersion. *Ω* reached 40 meV at 110 K (Figure 7e), and stable strong coupling persisted across the entire temperature range (Figure 7f). Tailoring the exciton‐photon detuning with temperature (negative at 130 K, near‐zero at 210 K, positive at 230 K) provided flexible control of the exciton‐polaritons. As the temperature increased, the Hopfield coefficients (Figure 7g) show that at small incidence angles, the LPBs transition from photon‐like to mixed and then to exciton‐like. This work overcomes the limitations of conventional systems, realizing controllable modulation of polariton composition, providing new possibilities for exploring important quantum phenomena such as Bose‐Einstein condensation (BEC) [38, 84, 85] and superfluidity [86]. Building on this research, Herira et al. [87]  studied the evolution of exciton‐photon detuning in a WS_2_‐microcavity system from 130 to 230 K. They clarified that detuning modulates polariton dispersion, composition, radiative lifetime, and scattering rate, and confirmed that longitudinal optical phonon‐assisted scattering dominates polariton relaxation, providing theoretical support for thermal control of polaritons and related quantum photonic devices. In addition to the control of coupling strength, Liu et al. [33] used the strong temperature dependence of WS_2_ excitons and the thermal stability of plasmonic nanocavity resonances to thermally optimize plasmon‐exciton coupling for two‐photon upconversion. The PL peak position of the exciton in WS_2_ significantly red‐shifted with increasing temperature (Figure 7h), while the scattering resonance peak of the plasmonic nano‐cavity (the yellow curve in Figure 7i) hardly changed with temperature. The results (Figure 7j, upper panel) show that the average two‐photon upconversion enhancement factor at 350 K was about 25% higher than at 300 K (room temperature), and the effective enhancement factor exceeded 3000. Power‐dependent measurements (Figure 7j, lower panel) showed that the upconversion intensities of both coupled WS_2_ (orange) and pure WS_2_ (blue) follow a quadratic power law *I*∝*P* ^2^. This confirms that the upconversion mechanism remains two‐photon absorption even after temperature increases, and that the enhancement arises solely from improved spectral matching, offering a simple route for actively controlling nonlinear luminescence in 2D excitons.

The advantage of thermal tuning lies in its operational simplicity and broad tuning range. By exploiting the strong temperature sensitivity of TMD excitons, the detuning parameter can be continuously swept over a wide energy range without requiring additional structural design or external fields. From a device perspective, however, the limitations of thermal tuning are rather evident. First, its dynamic response is slow, being governed by overall heat capacity and thermal diffusion processes, which makes truly high‐speed modulation difficult to achieve. Second, temperature variations not only shift the exciton energy but also simultaneously introduce multiple side effects, such as enhanced phonon scattering, linewidth broadening, and increased nonradiative recombination, rendering the system response difficult to control with a single parameter. Moreover, prolonged thermal cycling may lead to interfacial stress accumulation, thereby compromising device lifetime and repeatability.

### 3.4 All‐Optical Tuning

As a non‐contact and ultrafast modulation approach, all‐optical tuning exploits the nonlinear interaction between ultrafast laser pulses and matter. By varying the exciton density, this technique controls polariton energy shifts and coupling strength without requiring complex electrodes or mechanical structures. Precise parameter control tailors the exciton density, which controls exciton‐cavity coupling via nonlinear effects such as exciton–exciton and exciton‐photon interactions [88, 89].

**FIGURE 8:**
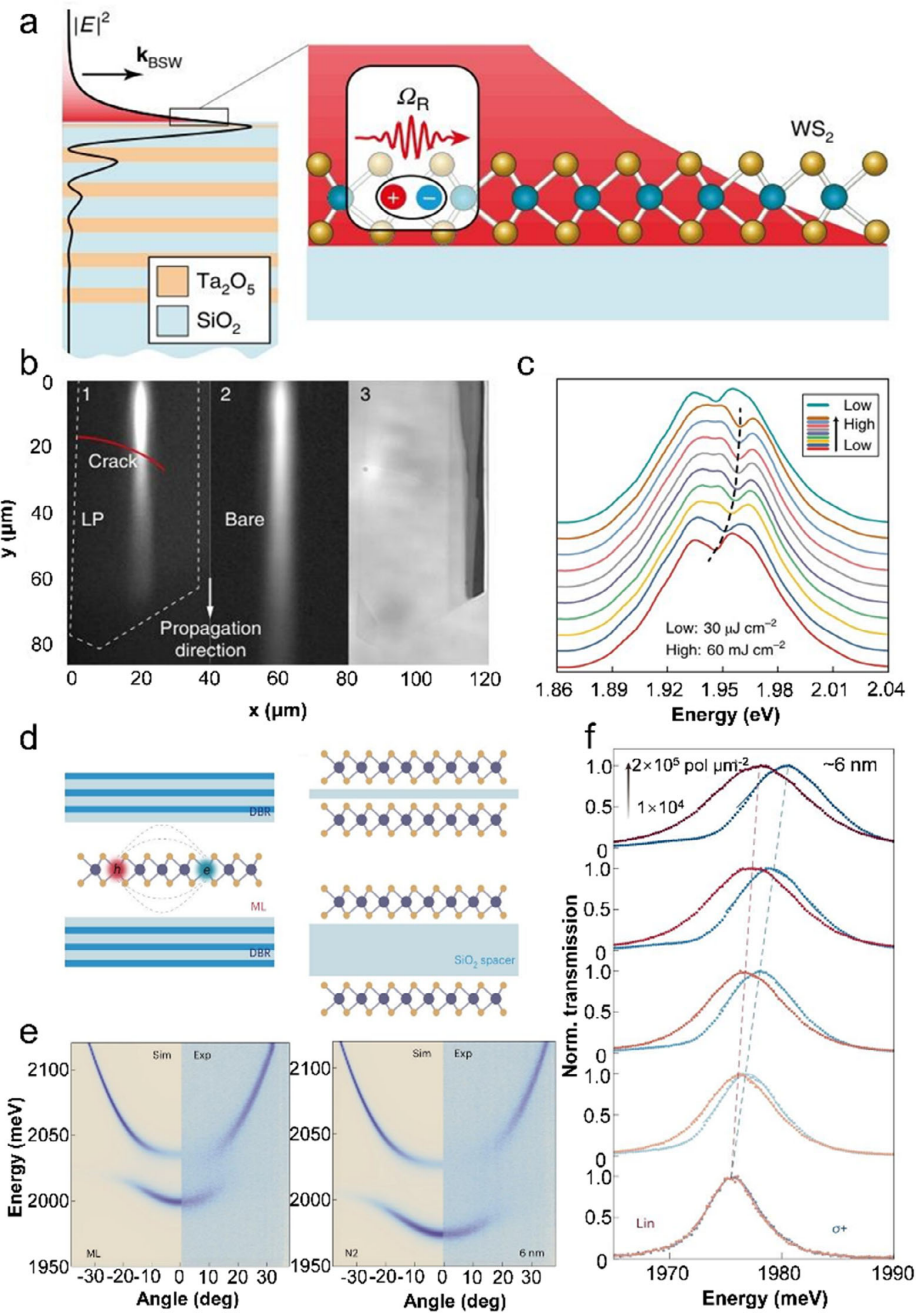
(a) Schematic of the dielectric stack supporting BSW polaritons in monolayer WS_2_. Black curve shows the electric field profile of the bare mode at the wavelength of the A exciton band. Inset: coupling of the enhanced electric field at the surface of the stack to the in‐plane excitons in the monolayer. (b) Resonant propagation at a wavelength of 645 nm for the LPB (exciton fraction of 10%) and bare modes, shown in panels 1 and 2, respectively. Corresponding propagation constants are 20.6 ± 0.1 µm and 21.1 ± 0.1 µm. Flake boundaries are indicated by white dashed lines. A small crack, indicated by a solid red line in panel 1, has very little impact on propagation, possibly due to the high photonic content of the mode. Panel 3 shows a micrograph of the monolayer in reflectance where the crack and boundaries can be seen. (c) LPB spectra as functions of the pump fluence at different pump energy densities (Reprinted/ adapted with permission [34] 2018, Springer Nature). (d) Schematic of the TMDs‐based microcavities, consisting of monolayer WS_2_ sandwiched between two DBRs (left) and schematic of double‐layer WS_2_ samples with different thicknesses of spacer layer, showing the thin spacer case (right). (e) Angle‐resolved reflectivity map of monolayer (left), double‐layer sample with the spacer of 6 nm (right). (f) Normalized transmission spectra of the double‐layer microcavity (spacer of 6 nm) with the circular (blue dots) and linear (red dots) polarized excitation of the LPB ground state (Reprinted/ adapted with permission [101] 2025, Springer Nature).

Modulating the pump power is the most common all‐optical tuning method. High‐power optical pumping generates high‐density excitons in TMDs, which enhances Coulomb interactions and induces nonlinear optical effects. The most characteristic effect is the blueshift of the polariton energy. At low densities, the interaction between excitons is weak. However, at high densities, the exciton–exciton scattering process is significantly enhanced, and its scattering rate can be expressed as [90]

(13) $$\gamma_{XX}\propto n_{X}^{2}\cdot {\left|M\right|}^{2},$$

where *n_X_* is the exciton density, and *M* is the average scattering matrix element. Exciton–exciton scattering directly leads to a blueshift in the exciton energy. This blueshift further modifies the exciton‐photon detuning. Meanwhile, high‐density excitons generate a dynamic polarization field that screens the electron–hole Coulomb interaction. This screening effect will reduce the binding energy of the exciton and change its wave function, thereby affecting the oscillator strength. Furthermore, the high‐density electron–hole pairs will alter the energy band structure of the semiconductor. Specifically, electron exchange‐correlation interactions reduce the bandgap [91]. The bandgap reduction and the change in exciton binding energy together determine the net shift of the exciton resonance. The final blueshift of polaritons is the result of the competition among these different effects [92, 93, 94, 95, 96]. By varying the pump power, the exciton density can be continuously tuned, enabling precise control over the magnitude of the polariton blueshift and the variation of energy detuning between excitons and cavity modes. This enables dynamic switching between strong and weak coupling and continuous modulation of the coupling strength. Barachati et al. [34] achieved strong coupling between excitons of a WS_2_​ monolayer and a Bloch surface wave (BSW) propagating at the air‐dielectric interface of a Bragg mirror (Figure 8a). They overcame the limitations of conventional structures, enabling long‐range polariton propagation exceeding 60 µm within the monolayer (Figure 8b). Figure 8c shows the full power sweep, with a maximum time‐averaged blueshift of 12.9 ± 0.5 meV. This blueshift is reversible, confirming the feasibility of precise power‐modulated control. The simple dielectric structure supporting strong coupling between excitons and propagating BSW offers a promising platform for room‐temperature interacting polariton fluids, enabling long propagation distances, reduced losses, and compatibility with multifunctional polaritonic circuits.

TMDs possess unique spin‐valley properties. Broken inversion symmetry combined with strong spin‐orbit coupling leads to the selective coupling of K/K' valley excitons with circularly polarized light of specific helicity. This enables polarization state modulation and extends the scope of all‐optical tuning [40, 97, 98, 99, 100]. Zhao et al. [101] achieved nonlinear control of spin‐layer‐locked polaritons at room temperature, revealing the cooperative modulation mechanism of polarization state and coupling strength. Both WS_2_ monolayer and bilayer microcavities are encapsulated between DBRs (Figure 8d). Angle‐resolved reflectivity confirms exciton‐polariton formation, with *Ω* values of approximately 34 meV (monolayer) and 52 meV (bilayer) (Figure 8e). As illustrated in Figure 8f, at low excitation density, the transmission signals for circular polarization (blue curve) and linear polarization (red curve) are indistinguishable. However, as the excitation density increases, the blueshift under circularly polarized excitation becomes more pronounced than that under linearly polarized excitation. This difference indicates the efficient spin‐dependent interactions in polaritons within the TMD bilayer. The phenomenon originates from spin‐dependent exciton–exciton Coulomb interactions dominating the nonlinear process, which suppresses spin‐independent polariton‐phonon coupling. In contrast, the WS_2_ monolayer exhibits a spin‐insensitive blueshift due to the absence of interlayer coupling, further verifying the influence of layer structure on the spin‐dependent coupling. Similarly, Hu et al. [100] utilized a SiO_2_ microsphere cavity coupled with a WS_2_/WSe_2_ heterostructure to selectively enhance the emission of spin‐triplet interlayer excitons, which possess an out‐of‐plane dipole moment contribution as high as 92%, through the Purcell effect of the cavity (while singlet excitons have only a 69% out‐of‐plane component). By adjusting the excitation power, they achieved reversible switching between singlet and triplet exciton emission. Moreover, leveraging the residual valley polarization of the triplet excitons, they demonstrated an all‐optical valley polarization switch with an on/off ratio of 35, setting a record in this field and providing a concise and efficient scheme for all‐optical valleytronic devices. This work simultaneously modulates exciton spin population and coupling strength via polarization control, extending all‐optical tuning from the energy domain to the spin domain.

All‐optical tuning, by virtue of its ultrafast response and contactless nature, exhibits unique advantages in polariton systems and is particularly suited for femtosecond switching, transient strong‐coupling modulation, and studies of nonequilibrium polariton dynamics. However, the bottlenecks of this approach lie primarily in thermal effects and stability issues. High‐power pumping inevitably generates local heating, causing the system to be simultaneously influenced by a mixture of thermal and nonlinear tuning, which reduces the selectivity of the control. Moreover, at high excitation densities, complex many‐body interactions render the system difficult to model precisely. Another critical limitation concerns the issue of persistence: most all‐optical tuning schemes are transient, with the system returning to its original state once the pump is removed, which makes them unsuitable for steady‐state logic devices or photonic circuits that require long‐term operation.

In summary, the four tuning strategies‐piezoelectric, electrical, thermal, and all‐optical modulation‐differ markedly in physical mechanisms, controllable degrees of freedom, performance metrics, and applicable scenarios, each offering distinct advantages and facing inherent limitations. For intuitive comparison, Table 1 summarizes their key features. Piezoelectric tuning provides a wide tuning range, high precision, and non‐invasive control, but it usually requires complex mechanical structures, exhibits relatively slow response, and remains challenging to miniaturize. Electrical tuning is highly compatible with FET technology, programmable, and suitable for on‐chip integration, although its performance can be sensitive to interface quality and carrier‐induced losses. Thermal tuning features simple implementation and does not require external electrodes or optical paths, but it suffers from slow response, global perturbation, and challenges in thermal management. All‐optical modulation enables ultrafast, contact‐free operation, yet it is often limited by a relatively narrow tuning range and the potential risk of optical damage under high pump fluence. Notably, continued advances in material quality, nanofabrication techniques, and emerging modulation mechanisms are expected to further extend the performance upper bounds of these approaches. Collectively, these four control strategies establish a comprehensive framework for full‐dimensional dynamic regulation of exciton‐photon coupling in TMD microcavity systems, offering flexible pathways for polaritonic device design across diverse application scenarios.

**TABLE 1:** Comparison of key performances of four dynamic tuning methods.

Aspect	Piezoelectric	Electrical	Thermal	All‐optical
Primary mechanism	Cavity‐length modulation	Carrier density/QCSE	Exciton energy shift via temperature	Exciton density induced nonlinear response
Tuning Speed	ms‐s level	ns‐ms level	ms‐s level	fs‐ps level
Reversibility	Reversible	Reversible	Reversible but with thermal hysteresis	Reversible
Non‐invasiveness	No direct modification of the active material	Carrier injection may introduce additional disorder	No external field required, but thermal stress is unavoidable	Electrode‐free operation, although high optical power may induce material degradation
Integration Compatibility	Requires precise piezoelectric mechanical structure and large volume	Compatible with FET technology, suitable for on‐chip integration	Requires integrated heating/cooling components, increasing system complexity	Requires external light source

## 4 Applications

### 4.1 All‐Optical Switches

**FIGURE 9:**
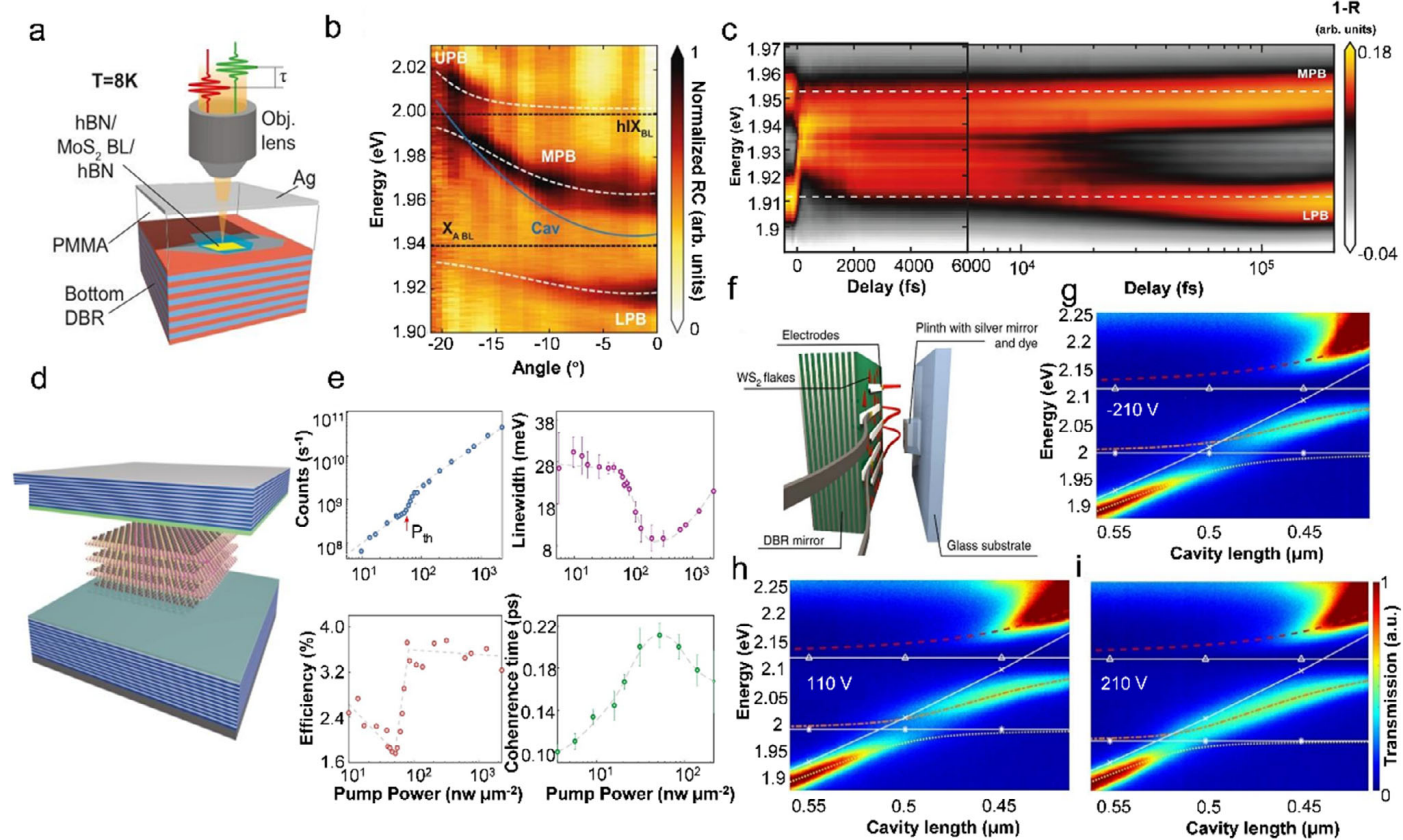
Ultrafast switch based on strong coupling in a bilayer MoS_2_ microcavity: (a) Sketch of the bilayer MoS_2_ microcavity structure measured by pump‐probe spectro‐microscopy. (b) Color map of the angle‐resolved reflectance contrast spectra of a microcavity embedding a bilayer MoS_2_ in the strong coupling regime. The coupled oscillators model fit (white dashed lines) and the cavity mode dispersion (blue line) are shown in overlay. (c) Color map of the 1‐R spectra of the microcavity as a function of the pump‐probe delay showing the ultrafast collapse and later revival of the middle polariton branch (MPB) and LPB (white dashed lines) (Reprinted/ adapted with permission [44] 2025, Springer Nature). Polariton laser based on a trilayer WS_2_ homostructure: (d) Schematic of the device structure. (e) Top‐left: Variation of PL photon emission with pump power. The two dashed lines show a linear relationship, and the red arrow denotes the *P_th_*. Top‐right: Relationship between polariton laser line width and pump power. Bottom‐left: Efficiency of the polariton laser as a function of pump power. Bottom‐right: Coherence time of the laser compared to the pump power (Reprinted/ adapted with permission [108] 2024, American Chemical Society). Electrically controlled polaritonic device based on monolayer WS_2_ and J‐aggregate dye TDBC: (f) Sketch of the two mirrors with silver electrodes on the surface of the DBR giving electrical tunability within the cavity. (g–i) Successive transmission spectra of hybrid WS_2_‐TDBC microcavity for decreasing cavity length from left to right and different applied voltages of −210, 110, and 210 V, respectively. The white, continuous lines correspond to the uncoupled energies of Frenkel‐exciton (TDBC, triangles), Wannier‐Mott exciton (WS_2_, stars), and cavity mode (crosses). The dashed lines in color show the dispersion for the coupled system consisting of the three polariton branches, LPB (yellow), MPB (orange), and UPB (red) (Reprinted/ adapted with permission [45] 2017, Springer Nature).

All‐optical switches are among the most promising device platforms for TMD polariton platforms because they exploit the intrinsic optical nonlinearity and ultrafast response of exciton‐polaritons for high‐speed signal modulation [102, 103]. In microcavity‐integrated TMD systems, ultrafast optical pumping can transiently reshape the exciton resonance, polariton dispersion, and even the strong‐coupling condition, thereby enabling low‐energy and high‐speed optical switching. These features make TMD microcavity polaritons attractive for integrated photonic logic and reconfigurable optical signal processing [5, 104]. The transient switching of the light‐matter coupling state through dynamic modulation is a key mechanism for improving the performance of all‐optical switches. Taking the MoS_2_ bilayer microcavity as an example, by leveraging its strong optical nonlinearity, ultrafast exciton relaxation, and interlayer/intralayer exciton interactions, reversible switching between strong coupling and weak coupling can be achieved on the femtosecond time scale. Under femtosecond pumping, the polariton branches transiently collapse into a weakly coupled cavity mode, accompanied by the closing of the polaritonic gap of up to 55 meV (Figure 9a–c). This mechanism leads to a switching threshold below 4 pJ per pulse, a room‐temperature operating threshold of 1.8 pJ, and a switching speed of 250 GHz, with a theoretical limit of up to 1 THz. The bilayer configuration also improves the extinction ratio to 7.5 dB.

Compared with conventional semiconductor optical switches, dynamically modulated TMD microcavity polaritons combine strong optical nonlinearity, ultrafast exciton dynamics, and enhanced light‐matter interaction, enabling low‐power operation without relying on ultrahigh‐Q cavities. These unique advantages not only improve switching speed and energy efficiency but also provide greater design flexibility for integrated photonic circuits. Therefore, microcavity‐based dynamic modulation represents a promising strategy for realizing compact, low‐power, and reconfigurable all‐optical information processing systems.

### 4.2 TMD‐Based Polariton Lasers

Polariton lasers are promising coherent light sources that rely on bosonic final‐state stimulation and the macroscopic occupation of lower‐polariton states, without requiring population inversion as in conventional lasers [84, 85, 105, 106, 107]. Owing to their low threshold, high coherence, and hybrid light‐matter nature, they hold considerable promise for integrated photonics and quantum optical technologies. By tailoring the exciton‐photon detuning, polariton dispersion, oscillator strength, and relaxation pathways, optical microcavities provide an effective route to optimizing the threshold, efficiency, coherence, and operating temperature of TMD‐based polariton lasers. Dynamic modulation of oscillator strength is one of the key strategies for achieving an ultralow‐threshold laser. As shown in Figure 9d,e, in a multilayer van der Waals heterostructure, by introducing dielectric spacer layers to separate adjacent TMD monolayers, the oscillator strength of excitons can be effectively enhanced, and the resonance condition between the cavity mode and the excitation transition can be optimized. This modulation method makes it possible for efficient polariton condensation near resonance, thereby achieving an ultralow laser threshold of 59 nW µm^−^^2^ and a laser efficiency of 3.82%. At the same time, dynamic modulation also significantly improves the coherence performance of the device: once the pump power exceeds the laser threshold, the coherence time can be extended from 100 to 211 fs [108]. In addition to the control of oscillator strength, the exciton localization provides another dynamic control path for a low‐threshold polariton laser. In the moiré heterostructure within the tunable microcavity, the exciton localization induced by the moiré potential can enhance the nonlinear interaction between polaritons. By changing the spatial localization characteristics of excitons to optimize the nonlinear response of polaritons, the laser threshold can be further reduced, providing a complementary physical idea for the design of polariton lasers [109]. These representative studies demonstrate that dynamic modulation can improve laser performance through different physical mechanisms. Compared with conventional static cavity optimization, dynamic modulation offers additional degrees of freedom for actively tailoring oscillator strength, exciton localization, and relaxation pathways in real time. This flexibility enables simultaneous optimization of lasing threshold, coherence, and operating stability, which is difficult to achieve through structural design alone. Such capabilities make dynamically tunable TMD polariton lasers particularly attractive for integrated coherent light sources and programmable photonic devices.

### 4.3 Electrically Controlled Polaritonic Devices

The robust room‐temperature excitonic resonances of TMD make them an attractive platform for electrically reconfigurable polaritonic devices. When integrated with tunable microcavities, these materials not only support strong exciton‐photon coupling but also enable in situ electrical control over the hybrid light‐matter states. Such systems offer opportunities for electrically tunable polariton composition, transport, and nonlinear optical response. The core advantage of electrical modulation lies in its ability to continuously and reversibly alter the exciton components of the polaritonic state. By integrating multiple exciton species into a microcavity and applying an external electric field, the resonance and absorption intensities of each exciton can be continuously controlled (Figure 9f–i). When multiple excitons coexist in the microcavity, the hybrid polaritonic mode becomes highly sensitive to the external electric field, and the exciton components can undergo significant redistribution in response to the electric field. This electrostatic component modulation directly enables the in situ tunability of the effective mass, interaction strength, and transport behavior of the polaritons, making it possible to switch multiple functions within a single device [45]. Although excessive charge‐induced disorder and optical loss remain important challenges, electrical modulation still offers several unique advantages. Electrical modulation provides continuous, reversible, and highly compatible control using mature electronic technologies, making it more suitable for large‐scale integration. The ability to dynamically manipulate polariton composition and transport within a single device further expands the functionality of polaritonic circuits, offering promising opportunities for multifunctional optoelectronic devices, programmable photonic chips, and electrically reconfigurable quantum photonic systems.

Overall, although the application scenarios differ, microcavity‐based dynamic modulation provides several common advantages across all TMD polaritonic devices, including real‐time and reversible tunability, enhanced light‐matter interaction, reduced power consumption, and improved device functionality. Unlike conventional static cavity engineering, dynamic modulation enables active optimization of polariton properties according to different operational requirements, thereby greatly increasing device flexibility and adaptability. These unique characteristics make dynamically tunable TMD microcavities a promising platform for next‐generation integrated photonics, low‐power optoelectronics, optical information processing, and quantum photonic technologies.

## 5 Conclusions and Outlook

This review summarizes recent progress in the microcavity‐enabled dynamic control of exciton‐polaritons in TMD, with an emphasis on the underlying mechanisms, major tuning strategies, and emerging device‐relevant functionalities. As atomically thin semiconductors, TMDs host tightly bound excitons with strong quantum confinement, large oscillator strength, and pronounced sensitivity to the surrounding dielectric and electronic environment. By providing strong photonic confinement and tunable light‐matter interaction, optical microcavities offer a versatile platform for engineering and dynamically controlling hybrid exciton‐photon states. The four modulation strategies have distinct characteristics and complementary advantages. Piezoelectric tuning primarily tailors the exciton‐photon detuning, mode structure, and photonic confinement through cavity‐length variation or geometrical reconfiguration, featuring simple operation and a wide tuning range. Electrical tuning enables reversible in situ control over exciton resonance, oscillator strength, carrier density, and even multi‐body polaritonic states through electrostatic doping and the quantum‐confined Stark effect. Thermal tuning provides a convenient and stable route to continuously scan exciton‐photon detuning and phonon‐assisted relaxation processes, although it is intrinsically slow and globally perturbative. All‐optical tuning enables contact‐free control on ultrafast timescales, avoids direct interference from electrodes or mechanical actuation, and is particularly attractive for high‐speed and minimally invasive polaritonic modulation. Collectively, these advances have established a solid basis for the development of functional excitonic and polaritonic devices based on TMD microcavities.

Overall, substantial progress has been made in the dynamic control of excitonic and polaritonic states in TMD microcavities, spanning both fundamental mechanisms and proof‐of‐principle device demonstrations. However, several key challenges still hinder the transition of TMD microcavity polariton systems from laboratory studies to practical applications. First, material and interface quality remain major bottlenecks, as interfacial charge transfer and defect scattering at the TMD‐microcavity interface shorten exciton coherence times and compromise device stability. Second, device‐level integration is still limited: the current degree of multi‐field synergistic control remains insufficient to satisfy the demands of complex and multidimensional functional devices. Third, scalable and compatible fabrication technologies for large‐area, high‐quality TMD films and microcavity architectures are still not sufficiently mature, which restricts reproducibility and practical integration. In addition, data‐driven cavity design and machine learning‐assisted parameter optimization may accelerate the identification of device architectures and operating conditions for targeted polaritonic functionalities. With continued advances in material quality, cavity engineering, and active control strategies, TMD microcavity polaritons are expected to evolve from proof‐of‐concept platforms into reconfigurable building blocks for next‐generation integrated optoelectronic, valleytronic, and quantum photonic devices.

## Conflicts of Interest

The authors declare no conflicts of interest.

## Data Availability

Data sharing not applicable to this article as no datasets were generated or analysed during the current study.
